# The Specific and Nonspecific Effects of Tai Chi and Its Possible Central Responses: A Protocol of Neuroimaging Study

**DOI:** 10.1155/2021/8883460

**Published:** 2021-02-19

**Authors:** Tianyu Liu, Yuke Teng, Sha Yang, Yuyi Guo, Tao Yin, Jingwen Chen, Rongtao Ying, Zhaoxuan He, Shuguang Yu, Jianwei Wu, Fang Zeng

**Affiliations:** ^1^School of Sport, Chengdu University of Traditional Chinese Medicine, Chengdu, Sichuan, China; ^2^Acupuncture and Brain Science Research Center, Chengdu University of Traditional Chinese Medicine, Chengdu, Sichuan, China; ^3^School of Acupuncture and Tuina, The 3rd Teaching Hospital, Chengdu University of Traditional Chinese Medicine, Chengdu, Sichuan, China; ^4^School of Chinese Classics, Chengdu University of Traditional Chinese Medicine, Chengdu, Sichuan, China

## Abstract

Tai Chi has been proven to be a safe and effective assistant therapy for healthcare and disease treatment. However, whether the adjuvant therapeutic effect of Tai Chi is general or disease-oriented remains uncertain. This trial focuses on exploring the specific and nonspecific effects of Tai Chi and its potential central responses. The results will deepen our understanding of the characteristics of Tai Chi exercise for adjuvant therapeutic effects and promote its application in the clinic. In this neuroimaging trial, 40 functional constipation (FC) patients and 40 healthy subjects (HS) will be recruited and will receive 10 weeks of Tai Chi exercise. The motor function, respiratory function, stool-related symptoms, quality of life, and emotional state of the participants will be evaluated at the baseline, the 5-week Tai Chi practice, and the end of practice. The potential changes in the heart rate variability and the cerebral function will be recorded by the 24 h dynamic electrocardiogram at the baseline and the functional magnetic resonance imaging at the end of practice. The possible correlations among the clinical variables, the heart rate variability, and the cerebral activity alterations in FC patients and HS will be analyzed. The healthcare and therapeutic effects of Tai Chi exercise might consist of the specific and nonspecific effects. This study provides not only a new perspective for understanding Tai Chi but also a new approach for investigating the mind-body exercise. This trial was registered in the Chinese Clinical Trial Registry (http://www.chictr.org.cn/showproj.aspx?proj=33243) on 28 November 2018 (registration number: ChiCTR1800019781; protocol version number: V1.0). This trial is currently in the stage of recruiting patients. The first patient was included on 1 December 2018. To date, 18 FC patients and 20 HS have been included. Recruitment will be completed in December 2020.

## 1. Introduction

Tai Chi is a traditional mind-body exercise, which originated from ancient China and widely spread all over the world. It is reported that there are 5 and 150 million Tai Chi practitioners in China and the whole world, respectively. Tai Chi has dual effects on healthcare and treatment. For healthy subjects (HS), the healthcare effects of Tai Chi in strengthening muscle strength [1], improving physical flexibility [2], enhancing the ability to balance and control [2], increasing vital capacity [3], reducing stress [4], and others have long been identified. For patients, the therapeutic effects of Tai Chi for treating multiple chronic diseases including osteoarthritis [5], hypertension [6], type 2 diabetes mellitus [7], coronary heart disease [8], and chronic obstructive pulmonary disease [9] have also been proven by a number of studies. These studies indicated that Tai Chi was widely involved in the prevention, treatment, and rehabilitation of various diseases. Does Tai Chi have different effects on the same organ/system in different practitioners? For example, a systematic review and meta-analysis showed that mind-body interventions, including Tai Chi, were effective in alleviating gastrointestinal symptoms and improving the quality of life (QOL) of patients [10]. Does Tai Chi have different gastrointestinal modulation effects between patients with gastrointestinal disorders and HS? In other words, is the gastrointestinal regulation of Tai Chi disease-oriented and influenced by individual physical condition? However, majority of the Tai Chi-related studies were performed either on patients or HS, and few studies were performed on both patients and HS to investigate the influence of physical conditions on the effect of Tai Chi.

On the basis of the characteristics of Tai Chi and previous researches, we predict that the effects of Tai Chi include two aspects: the relatively specific effects and nonspecific effects. The nonspecific effects mainly refer to the modulating effects on human motor function (muscle strength, physical flexibility, etc.) and respiratory function, especially vital capacity. The nonspecific effects might also manifest as maintaining the normal function of organs/systems, keeping the body in a relatively balanced and coordinated state. While the relatively specific effects of Tai Chi are disease-oriented, meaning that when the body is in a pathological state, Tai Chi practice might reduce the hyperactive function to improve the hypoactive function.

To test the hypothesis, we design this neuroimaging study. In this study, both functional constipation (FC) patients and HS were selected as participants to investigate the specific effects (gastrointestinal function) and nonspecific effects (motor function and respiratory function) of Tai Chi. FC is a common functional gastrointestinal disorder (FGID) with high prevalence. It is characterized by various constipation-related symptoms, including reduced defecation, defecation stress, hard stools, and uncomfortable abdominal muscle in the absence of evident organic or structural reasons for these symptoms. A randomized control trial indicated that Tai Chi practice could significantly improve the constipation symptom of patients with chronic FC [11].

This study aims to (1) investigate the nonspecific effects of Tai Chi by comparing the influence of Tai Chi practice on motor function and respiratory function between FC patients and HS using the lower-extremity muscle strength test, functional balance tests, and vital capacity test; (2) investigate the specific effects of Tai Chi from three aspects, including gastrointestinal symptom, autonomic nervous activity, and emotional state; and (3) explore the potential central responses of Tai Chi's specific and nonspecific effects by functional magnetic resonance imaging (fMRI).

## 2. Methods and Analysis

### 2.1. Study Design

**Figure 1:**
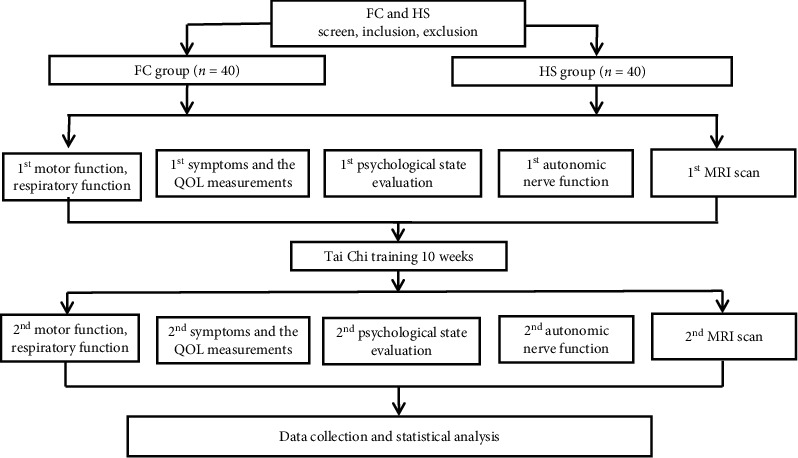
Study flowchart. This is a neuroimaging trial which focuses on specific and nonspecific effects of Tai Chi and its potential central responses. Totally, 40 eligible FC patients and 40 HS will be recruited. Fifteen participants in each group will be randomly selected to undergo functional magnetic resonance imaging (fMRI) scan.

The trial is designed as a neuroimaging trial that focuses on specific and nonspecific effects of Tai Chi and its potential central responses. A total of 40 FC patients and 40 HS will be recruited. Fifteen participants in each group will be randomly selected to undergo MRI scan. The study procedure is outlined in Figure 1.

### 2.2. Sample Size Calculation

We will investigate the therapeutic effects of Tai Chi for FC patients. According to our preliminary study from which we recruited 10 FC patients with the Tai Chi intervention, the mean improvement of weekly complete spontaneous bowel movements (CSBMs) of Tai Chi was 1.67 ± 0.89 times. A systematic review [12] focusing on the management of FC reported the mean improvement of weekly CSBMs of polyethylene glycol (the first-line medication of FC) was 1.8 times. We hypothesize that the therapeutic effects of Tai Chi are not less than the polyethylene glycol. Considering *α* = 0.05, 1 − *β* = 0.8, the study design required a sample size of 36 for each group with the one-sample noninferiority test [13], with a drop-out rate of 10%. A total of 40 patients with FC and 40 HS will be finally recruited.

(1) $$\begin{array}{c} N={\left(\sigma \frac{Z_{1-\alpha }+Z_{1-\beta }}{\mu -\mu o-\delta }\right)}^{2}. \end{array}$$

### 2.3. Patients with FC

Patients who match the inclusion criteria will be recruited. FC patients will be diagnosed by two gastroenterologists in the digestion department of the Hospital of CDUTCM, according to the Rome IV Diagnostic Criteria for FC [14]. Each FC patient will undergo a careful physical examination including a routine blood test, routine urine test, routine stool test, blood biochemical test (ALT, AST, BUN, Scr), transabdominal ultrasound, and dynamic electrocardiogram. The inclusion criteria and exclusion criteria of FC patients were as same as our previous study [15].

#### 2.3.1. Inclusion Criteria

Patients will be included in the study if they (1) reach the Rome IV Diagnostic Criteria for FC [14], (2) are right-handers and the age range is between 18 and 35, (3) have less than three CSBMs every week, (4) the Cleveland Constipation Score (CCS) score >10, and (5) provide a written informed consent form.

#### 2.3.2. Exclusion Criteria

Patients will be excluded from the study if they (1) are pregnant women, or plan to be pregnant within 3 months, or are breast-feeding women, (2) are incapable of sports, (3) have a history of head trauma and loss of consciousness, (4) have diabetes or serious cardiovascular, neurological, psychiatric, renal, or respiratory disease, (5) have moderate or serious depression and anxiety, (6) cannot keep silent for 20 minutes while lying down, (7) have any contraindications to fMRI scanning including the presence of metal stent, metal denture, or claustrophobia, (8) have taken other exercises (including meditation and yoga) that may improve constipation over 30 minutes every week in the last 3 months, (9) have taken gastrointestinal motility medicine, nonsteroidal anti-inflammatory medicine, and steroids in the last 15 days, (10) have received other treatments (including surgery, diet modification, biofeedback, or probiotics) in the last month, or (11) have participated in any other clinical trials in the past 3 months.

### 2.4. Healthy Subjects

Those who match the inclusion criteria will be recruited and will receive the same physical examination with the FC patients.

#### 2.4.1. Inclusion Criteria

Subjects will be included if they (1) are right-handers and aged 18–35 years, (2) have no abnormalities during the physical examinations, (3) have passed the National Student Physical Health Standard test, and (4) provide written informed consent.

#### 2.4.2. Exclusion Criteria

The exclusion criteria for HS are the same as the criteria for FC patients (see Section 2.3.2). Moreover, those who have possible organic diseases, psychological disorders, and gastrointestinal symptoms and signs are also excluded.

### 2.5. Recruitment Strategy

The participants will be recruited from the Chengdu University of Traditional Chinese Medicine (CDUTCM) by delivering leaflets inside the campus, posting advertisements in billboards, posting at Acupuncture and Brain Science Research Center and CDUTCM (http://cdutcm.edu.cn/) websites, and posting in our WeChat public account.

### 2.6. Intervention

The participants will undergo 40 sessions with 24-style Tai Chi. The exercise of Tai Chi will be performed four times a week (Monday, Tuesday, Thursday, and Friday) and will last for 10 weeks [15]. Each exercise session lasts for an hour, including 10-minute warm-up, 40-minute Tai Chi exercise, and 10-minute relaxation.

Before the formal intervention, participants were involved in motor learning and strength training under the guidance of two professional Tai Chi instructors. During the intervention, they exercise strictly under the 24-style Tai Chi exercise standard operating procedure (SOP), which was formulated by two Tai Chi experts. In each session of the exercise, the instant heart rate will be counted both at the pre- and posttrial, and the sports self-rating scale will also be filled out. Participants who did not come will be recorded. The training will be considered effective when the rating of perceived exertion (RPE) of the subjects reaches 4–6 levels in each session, and training times must not be less than 80% (32 times) of total training times.

During the treatment period, participants are usually not recommended to use concomitant care or interventions. However, if required (such as, aggravation of the patient's condition), participants will be permitted to use extra osmotic laxatives. The type and dosage of medication used should be recorded in the case report forms (CRFs).

### 2.7. Outcome Measurements

Measurements will be evaluated by independent assessors who have been trained prior to the study. All results will be recorded whether or not the participants completed the study. The outcome assessors will be independent of the research team and will not be told about group allocation, so as to ensure the object evaluations of the trial.

#### 2.7.1. General Information Collection

Same as our previous study [15], general information including the demographic data and vital signs will be collected. The demographic data include age, gender, education level, nationality, and body mass index (BMI). The vital signs include body temperature, heart rate, respiratory rate, and blood pressure. Among them, the BMI will be calculated at the baseline and the end of the invention, whereas the vital signs of each patient will be immediately recorded after every training.

According to our hypothesis, the nonspecific effects of Tai Chi include motor function, vital capacity, and psychological state regulation and the specific effects of Tai Chi for FC patients reflects in relieving gastrointestinal symptoms and modulating autonomic nerve function.

#### 2.7.2. Nonspecific Effects: Motor Function, Vital Capacity, and Psychological State

The lower-extremity muscle strength, functional balance tests, and the vital capacity test will be collected at the baseline, the median of intervention (after 5 weeks of exercise), and at the end of the intervention. The lower-extremity muscle strength will be measured using a hand-held isometric dynamometer (Micro FET3; Hoggan Health Industries, Draper, UT, United States). Functional balance tests include the Berg Balance Scale (BBS) [16], timed up-and-go (TUG) test, and functional reach test [17].

During the vital capacity test, the forced expiratory volume in one second (FEV1), forced vital capacity (FVC), and FEV1/FVC ratio will be measured using a Super Spiro spirometer (MicroMedical, Rochester, Kent, UK) in resting status. The peak expiratory flow rate (PEFR) will be assayed using a peak flow meter.

To assess the psychological state of all participants, we collected the following metrics at the baseline, the median of intervention, and the end of intervention: the self-rating depression scale (SDS) [18], the self-rating anxiety scale (SAS) [19], and the Eysenck Personality Questionnaire (EPQ) [20].

#### 2.7.3. Specific Effects: Gastrointestinal Symptoms and Autonomic Nerve Function

To evaluate the changes of the gastrointestinal symptoms and QOL of participants, we performed the following measurements at the baseline, the median of intervention, and the end of intervention: CCS [21], patient assessment of constipation symptoms (PAC-SYM) [22], patient assessment of constipation QOL questionnaire (PAC-QOL) [23], RPE [24], and MOS 36-item short-form health survey (SF-36) [25]. Also, the evaluation of constipation diary, including CSBMs per week, the fecal character, and the difficulty degree of defecation, will be measured once a week for 10 times.

To evaluate the autonomic nervous function, we selected the heart rate variability (HRV). All participants will be assessed during the 24-h HRV at the baseline and the end of the intervention. The metrics of HRV include standard deviation of NN intervals (SDNN), standard deviation of sequential 5-min RR interval means (SDANN), and root mean square successive difference (RMSSD). The device used is a dynamic electrocardiogram (ct-086S; BENE WARE, Hangzhou, China).

### 2.8. MRI Data Acquisition

MRI data will be collected on 15 participants in each group at the baseline and the end of intervention. The acquisition parameter will be consistent with our previous article [15].

The MRI scan includes three sequences: a high-resolution 3-dimensional T1-weighted imaging (3D-T1W1), a blood oxygenation level-dependent functional MRI (BOLD-fMRI), and a diffusion tensor imaging (DTI) sequence. Scanning will start in the morning after overnight fasting. Subjects who are selected will undergo MRI scan with a 3.0 T magnetic resonance scanner (Siemens, Germany). The 3D-T1WI scan parameters will be as follows: repetition time/echo time: 1900 ms/2.26 ms; slice thickness: 1 mm, slice number: 176, matrix size: 128 × 128, and the view field: 256 × 256 mm^2^. The BOLD-fMRI scanning parameters are as follows: repetition time/echo time: 2000 ms/30 ms; flip angle: 90°; slice number: 30; matrix size: 128 × 128; view field: 240 × 240 mm^2^; slice thickness: 5 mm; and total volume: 240. DTI data are as follows: view field: 240 × 240 mm^2^; repetition time/echo time: 6800 ms/93 ms; matrix size: 128 × 128; and slice thickness: 3 mm, seamless. Two diffusion-weighted sequences will be acquired using gradient values b: 1000 s/mm^2^ and b: 0, with diffusion-sensitizing gradients used in 64 noncollinear directions.

### 2.9. Data Management

The clinical data will be managed with printed and electronic CRFs. CRFs will be entered parallelly and will only be available to the outcome assessors. The Evidence-based Medicine Center of the CDUTCM will be responsible for monitoring the study and data every 3 months.

### 2.10. Statistical Analysis

#### 2.10.1. Clinical Data Analysis

Clinical data will be analyzed by independent statisticians who do not know the test procedure based on the principles of intention to treat (ITT) and per protocol. The statistical significance threshold has *p* value <0.05. The results of the participants who failed to complete the study will be considered no different from the baseline data in ITT analysis. All continuous variables will be presented as mean ± standard deviation. The categorical variables will be described in percentage (%). The clinical data in the two groups (FC group and HS group) will be compared with two-sample *t*-tests, and the comparisons of the baseline and after intervention in each group will be compared with a paired sample *t*-test. Nonparametric tests (Mann–Whitney *U* test) will be used to compare nonnormally distributed clinical data, and the *χ*^2^ test or Fisher's test will be used to compare categorical variables.

#### 2.10.2. Functional MRI Data Analysis

For fMRI scans, all preprocessing steps will be performed using DPABI software based on MATLAB. The main analytical methods include the amplitude of low-frequency fluctuation amplitude (ALFF) and seed-based functional connectivity.

After preprocessing the data, the ALFF will be calculated to compare the whole-brain spontaneous activity pattern before and after intervention in each group, as well as between patients with FC and HS following 10-week Tai Chi practices. The different regions obtained at ALFF analysis will be selected as the region of interest (ROI), also called seed, to perform the seed-based functional connectivity analysis and to explore the functional synchronization of ROI and other regions. The thresholds of *p* < 0.05 with a false discovery rate correlation will be applied to all analyses.

#### 2.10.3. Correlation Analysis

To investigate the associations between nonspecific effects and central responses under different physical conditions, we performed the correlation analysis between the clinical data, including motor function, respiratory function, psychological state, and cerebral function in patients with FC and HS. In order to further explain the regulation mechanism of Tai Chi on the brain-gut interaction disorder, the correlation analysis among gastrointestinal symptoms, heart rate variability, and brain function activities in FC patients will also be carried out.

#### 2.10.4. Safety Assessments

Adverse events might happen during Tai Chi practice, including strain, sprain, nausea, and dizziness. If any these adverse events occur, the instructor will take appropriate treatment according to clinician's advice and record the processing detail in the CRFs. The safety assessments will be monitored by the Ethics Committee.

The schedule of the study, including enrollment, interventions, assessments, and visits for participants, is shown in Table 1.

## 3. Discussion

As a popular physical–mind exercise, Tai Chi is widely used in the prevention, treatment, and rehabilitation of various diseases. On the basis of the characteristics and related studies on Tai Chi, we put forward the original hypothesis that Tai Chi practice can produce relative specific effects and nonspecific effects on the human body.

The main purposes of this study include two aspects. First, this study tries to explore the physiological and psychological existence of the specific and nonspecific effects of Tai Chi by comparing the differences in the motor function, respiratory function, gastrointestinal symptoms, psychological manifestations, and HRV between FC patients and HS after Tai Chi practice. Second, this study tries to explore the potential central mechanism of the specific and nonspecific effects of Tai Chi practice by analyzing the potential cerebral activity changes induced by Tai Chi and their correlation with clinical variables. The study will provide a new approach for investigating the mind-body exercise, and the results might deepen our knowledge about Tai Chi.

### 3.1. Specific and Nonspecific Effects of Tai Chi

Tai Chi is a complex sport that requires the coordination of spirit, breathing, postures, and movements. It can produce a significant impact on almost all human systems such as motor system, cardiovascular system, respiratory system, and digestive system. The impact is far beyond the physiological and psychological changes brought by a simple physical exercise. Thus, Tai Chi is not only widely used in public healthcare but also involved in disease treatment and rehabilitation.

On the basis of the characteristics of Tai Chi and related studies, we put forward that there are two types of effects of Tai Chi. One is the specific effect, which mainly indicates the therapeutic effects of Tai Chi practice on the pathological status and has obvious disease orientation. For instance, Tai Chi practice could produce a specific-regulating effect on the abnormal gastrointestinal (GI) function of patients with GI disorders, specifically improve the cognitive function of patients with Alzheimer's disease [26], and significantly relieve the depression symptoms of patients with depression [27]. A systematic review and meta-analysis demonstrated that Tai Chi and other mind-body interventions were effective in alleviating GI symptoms and improving several aspects of the disease-related QOL including interference with activity, body image, health worry, food avoidance, and social reaction [10].

The second aspect is the nonspecific effects, which mainly refers to the benefits for the motor system, respiratory system, and others. No matter Tai Chi practice or other forms of physical exercises can produce it. The nonspecific effects might be a promoting effect on the motor system and respiratory system for not only the patients but also the HS. For example, studies demonstrated that Tai Chi exercises could increase the muscle strength in the lower extremities, improve balance control, and reduce the risk of falls for both healthy elderly people [28, 29] and Parkinson's and stroke patients [30, 31]. However, it should be emphasized that the specific effects of Tai Chi are relative and conditional. The nonspecific effects are the more widely used and fundamental effect of Tai Chi.

### 3.2. Potential Central Responses of Tai Chi

As a typical physical and mental exercise, Tai Chi not only emphasizes physical movement but also requires the state of unity of body and mind. The positive effects of meditation and aerobic exercise on cognitive function, including memory and decision-making abilities, have been widely accepted [32, 33]. Recent studies further identified the influence of Tai Chi practice on the cerebral structure and function of practitioners [34]. For example, MRI studies showed that Tai Chi training evoked significant changes in the brain white matter network [35], brain volume [36], and spontaneous brain functional activities [37] in older people. These studies indicated that Tai Chi practice could influence not only the cerebral function but also the brain structure. It might be the central mechanism of the specific and nonspecific effects of Tai Chi.

In this study, FC patients will be enrolled to investigate the specific effects of Tai Chi-modulating GI function. FC, as a typical FGID, the dysfunction of the gut-brain axis plays an essential role in its pathogenesis [38]. Recent neuroimaging studies identified the abnormality in the cerebral function and structure in FC patients compared with the healthy controls. For example, an fMRI study demonstrated that FC patients showed significant differences in baseline brain activities in several brain regions implicated in emotional process modulation, somatic and sensory processing, and motor control regions [39]. Considering the existence of cerebral structural and functional abnormalities in FC patients, it is feasible to explore the mechanism of Tai Chi's specific effects on GI function of FC patients.

In this study, fMRI will be selected as the neuroimaging technique to investigate the cerebral activity changes elicited by Tai Chi practice. fMRI is the most commonly used brain imaging technique and has been widely used in the studies on the central mechanism of Tai Chi intervention [35, 40, 41]. For example, using fMRI, people found that influencing the resting-state functional connectivity alteration between the cognitive control network and rostral anterior cingulate cortex and medial prefrontal cortex might be the central mechanism of Tai Chi intervention for fibromyalgia [41]. These studies indicated that it was reliable to use fMRI to explore the central mechanism of Tai Chi intervention for FC.

Furthermore, to ensure the reliability and repeatability of the results, we will establish a strict quality control program, which includes the participant selection, the SOP of Tai Chi practice, the SOP of neuroimaging scan, and the outcome measures. For example, for the selection of participants, the freshmen who never practice Tai Chi and have no physical exercise frequently will be taken into consideration. In the implementation of Tai Chi intervention, the SOP of 24-style Tai Chi is established, which covers the requirement on the movements, breathing, and ideas of the participant.

## 4. Conclusions

In conclusion, Tai Chi is a safe and effective assistant therapy for many diseases. However, whether the therapeutic effects of Tai Chi are disease-oriented remains uncertain. This trial is the first to investigate the existence of the specific and nonspecific effects of Tai Chi and to explore their potential central responses. It will provide a new perspective for understanding Tai Chi and a new approach for studying mind-body exercise.

## Figures and Tables

**Table 1:** Study period.

	Study period					
	Enrollment	Allocation	Postallocation			
Timepoint^*∗∗*^	−14 days	−7 days	0 day (baseline)	35 days (middle of intervention)	70 days (after intervention)	Etc.
Enrollment						
Eligibility screen	✗					
Informed consent	✗					
Demographics	✗					
Diagnosis	✗					
Past medical history	✗					
Physical examination		✗				
Allocation		✗				

Interventions						
Group A (FC patients)			✗	✗	✗	
Group B (HS)			✗	✗	✗	

Assessments						
BMI		✗			✗	
HRV		✗			✗	
Lower-extremity muscle			✗	✗	✗	
Functional balance			✗	✗	✗	
Vital capacity			✗	✗	✗	
Exercise self-rating scale			✗	✗	✗	
CCS			✗	✗	✗	
PAC-QOL			✗	✗	✗	
PAC-SYM			✗	✗	✗	
Stool diary			✗	✗	✗	
SDS, SAS			✗	✗	✗	
EPQ			✗	✗	✗	
SF-36			✗	✗	✗	
MRI			✗		✗	

Safety observation						
Blood routine test			✗			
Urine routine test			✗			
Stool routine test			✗			
Adverse events					✗	

This is a neuroimaging trial that includes a 2-week baseline period and a 10-week treatment period. In the baseline period, recruited patients will be screened according to the inclusion criteria and exclusion criteria, and then, eligible FC patients and HS will sign an informed consent form and receive a physical examination. After allocation, the FC patients and HS will be recruited and will receive 10 weeks of Tai Chi exercise. Schedule of enrollment, interventions, and assessments: at the baseline, the median of intervention (5 weeks of exercise), and the end of the intervention (10 weeks of exercise). Among them, lower-extremity muscle strength was measured using a hand-held isometric dynamometer (Micro FET3; Hoggan Health Industries). Functional balance tests include BBS, TUG test, and functional reach test. During the vital capacity test, FEV1, FVC, and FEV1/FVC ratio were measured using a MicroMedical Super Spiro spirometer in resting status. PEFR was assayed using a peak flow meter. The stool diary, CCS, and PAC-SYM will be used to evaluate the clinical efficacy of different interventions; the PAC-QOL will be used to assess the health-related QOL; the SDS, SAS, EPQ, and Mini-Mental State Examinations will be used to consider the effect of psychological factors on the patients' symptoms. All participants will be assessed during the 24 h HRV to evaluate the autonomic nervous function at the baseline and the end of the 10-week intervention. fMRI scans will be performed to detect the cerebral functional changes in 15 patients in each group both at the baseline and the end of the intervention.

## Data Availability

This is a protocol for a clinical trial, and no original data are included.

## Abbreviations

HS: Healthy subject
FC: Functional constipation
FGID: Functional gastrointestinal disease
QOL: Quality of life
GI: Gastrointestinal
fMRI: Functional magnetic resonance imaging
CDUTCM: Chengdu University of Traditional Chinese Medicine
CSBMs: Complete spontaneous bowel movements
CCS: Cleveland Constipation Score
RPE: Rating of perceived exertion
PAC-SYM: Patient Assessment of Constipation Symptom
PAC-QOL: Patient Assessment of Constipation Quality of Life Questionnaire
RPE: Rating of perceived exertion
SF-36: The MOS 36-item short-form health survey
BBS: Berg Balance Scale
TUG: Timed up-and-go
FEV1: Forced expiratory volume in one second
FVC: Forced vital capacity
SDS: Self-Rating Depression Scale
SAS: Self-Rating Anxiety Scale
EPQ: Eysenck Personality Questionnaires
HRV: Heart rate variability
3D-T1WI: 3-dimensional T1-weighted imaging
BOLD-fMRI: Blood oxygenation level-dependent fMRI
DTI: Diffusion tensor imaging
CRF: Case report form
ITT: Intention to treat
PP: Per protocol
ReHo: Regional homogeneity
ALFF: Amplitude of low-frequency fluctuation amplitude.
